# Cholesterol is Inefficiently Converted to Cholesteryl Esters in the Blood of Cardiovascular Disease Patients

**DOI:** 10.1038/s41598-018-33116-4

**Published:** 2018-10-03

**Authors:** Mathias J. Gerl, Winchil L. C. Vaz, Neuza Domingues, Christian Klose, Michal A. Surma, Júlio L. Sampaio, Manuel S. Almeida, Gustavo Rodrigues, Pedro Araújo-Gonçalves, Jorge Ferreira, Claudia Borbinha, João Pedro Marto, Miguel Viana-Baptista, Kai Simons, Otilia V. Vieira

**Affiliations:** 1 Lipotype GmbH, Tatzberg 47, 01307 Dresden, Germany; 2 CEDOC, NOVA Medical School, Faculdade de Ciências Médicas, Universidade NOVA de Lisboa, 1169-056 Lisboa, Portugal; 3 Hospital Santa Cruz, Centro Hospitalar de Lisboa Ocidental, Av. Prof. Dr. Reinaldo dos Santos, 2790-134 Carnaxide, Portugal; 4 Neurology Department, Hospital Egas Moniz, Centro Hospitalar de Lisboa Ocidental, Rua da Junqueira 126, 1349-019 Lisboa, Portugal; 5 Present Address: Centre de Recherche, Institut Curie, 26 rue d’Ulm, 75248 Paris Cedex 05, France

## Abstract

Shotgun lipidomic analysis of 203 lipids in 13 lipid classes performed on blood plasma of donors who had just suffered an acute coronary syndrome (ACS, *n* = 74), or an ischemic stroke (IS, *n* = 21), or who suffer from stable angina pectoris (SAP, *n* = 78), and an age-matched control cohort (*n* = 52), showed some of the highest inter-lipid class correlations between cholesteryl esters (CE) and phosphatidylcholines (PC) sharing a common fatty acid. The concentration of lysophospatidylcholine (LPC) and ratios of concentrations of CE to free cholesterol (Chol) were also lower in the CVD cohorts than in the control cohort, indicating a deficient conversion of Chol to CE in the blood plasma in the CVD subjects. A non-equilibrium reaction quotient, *Q*′, describing the global homeostasis of cholesterol as manifested in the blood plasma was shown to have a value in the CVD cohorts (*Q*′_ACS_ = 0.217 ± 0.084; *Q*′_IS_ = 0.201 ± 0.084; *Q*′_SAP_ = 0.220 ± 0.071) that was about one third less than in the control cohort (*Q*′_Control_ = 0.320 ± 0.095, *p* < 1 × 10^−4^), suggesting its potential use as a rapid predictive/diagnostic measure of CVD-related irregularities in cholesterol homeostasis.

## Introduction

Cholesterol in the blood plasma compartment exists in two forms, free cholesterol (Chol) and cholesteryl esters (CE), both of which are constituents of circulating lipoproteins (chylomicrons (CM), Very Low Density Lipoproteins (VLDL), Intermediate Density Lipoproteins (IDL), Low Density Lipoproteins (LDL), and High Density Lipoproteins (HDL)). Of these lipoprotein particles, CM originate at the enterocytes of the small intestine^1^ whereas VLDL have their origin in the liver and are converted to IDL and LDL^2,3^. Esterification of Chol to CE in both intestine and liver cells occurs in the endoplasmic reticulum and is catalyzed by an acylCoA-cholesteryl-acyl transferase (ACAT). All lipids that constitute CM, VLDL, IDL and LDL are destined for delivery to peripheral tissues, failing which they are returned to the liver where they are reprocessed for excretion or recirculation.

HDL particles, on the other hand, are produced (mostly by the liver and intestine) as apolipoproteins either free of lipid or associated with a small amount of phospholipids (the so-called pre-β particles). Phospholipids and Chol are transferred from cells of the peripheral tissues mostly via cellular efflux pumps, or from core-lipid-depleted lipoproteins in the blood to nascent HDL to form discoid bilayers of phospholipids and cholesterol stabilized by apolipoproteins. The serum enzyme lecithin-cholesterol acyl transferase (LCAT) associates preferentially with discoid HDL and catalyzes transfer of the *sn*-2 fatty acid of phosphatidylcholines (PC) to Chol (both constituents of the same particle) to produce CE and lysophosphatidylcholines (LPC)^4^. Due to their hydrophobicity CE partition into the bilayer midplane of the discoidal HDL particles giving them a progressively more spherical shape (HDL_3_ particles). Fusion and remodeling processes convert the denser HDL_3_ to larger and less dense spherical HDL_2_ particles whose core CE are either exchanged for triglycerides with other lipoprotein particles or are taken up by the liver for excretion or recirculation, making HDL the main players in reverse cholesterol transport. Notwithstanding all this information, we lack an overall quantitative description of the physiological chemistry of HDL in the blood plasma^5–8^.

Blood cholesterol levels have long been used as indicators of cardiovascular disease (CVD) risk. The incidence of CVD has a well-established positive correlation with total cholesterol concentrations associated with LDL (LDL-C) and a negative correlation with total cholesterol concentrations associated with HDL (HDL-C)^9,10^. While both correlations have robust epidemiological bases and while the positive correlation of LDL-C with CVD risk is reasonably understood, the negative correlation of HDL-C with CVD risk is poorly understood from the biochemical and physiological perspectives^7,11–13^.

Quantitative mass spectrometry-based shotgun lipidomics of blood plasma^14^ allows us to obtain data at lipid molecular subspecies resolution, permitting a better understanding of the biochemistry involved. This approach minimizes experimental manipulation of the object of study (blood plasma, lipoproteins) which may, in principle, affect compositional characteristics of non-covalently associated constituents. Combination of this analytical approach with algorithms that describe reaction and tissue/plasma partitioning processes in the system could provide useful information that may be of relevance to clinical diagnostics.

We have compared lipidomic profiles of blood plasma obtained from donors who had suffered an acute coronary syndrome (ACS, including ST-elevation and non-ST-elevation myocardial infarction and unstable angina pectoris), donors with stable angina pectoris (SAP), donors who had suffered an ischemic stroke (IS), and an age-matched control cohort whose members had never sought clinical help for a CVD-related problem. Our results indicate that the CE/Chol ratio in the blood plasma is significantly lower in the CVD donors compared with the control group and we show that this may be attributed to a reduced activity of LCAT in the CVD patients, suggesting that inefficient conversion of free cholesterol to cholesteryl esters by LCAT may be a significant contributing factor to the inverse correlation of HDL-cholesterol with CVD risk.

## Results

### Blood Plasma Lipid Concentrations in Control and CVD Cohorts

Lipidomic analysis yielded the lipid concentrations in the blood plasma of the “Control” and “CVD” cohorts (Table S1). Table 1 lists the average concentrations of the lipids relevant to this work for each of the cohorts. Absolute differences in the plasma lipid concentrations of the different cohorts may have more than one cause. Reduction of total cholesterol (CE + Chol) concentrations by specific medication (statins and ezetimibe, for example) is well established. Decreases in lipid concentrations in the blood plasma have also been attributed to the so-called acute-phase response, although the exact values are disputed^15,16^. For the purpose of this work, however, the non-uniform changes in the average concentrations of the different lipid classes suggests that more than one mechanism may be responsible for the observation. Differences in the concentration ratios of biochemically interconvertible species (CE and Chol, or PC and LPC) must, however, have a physiological cause.

**Table 1:** Lipid concentrations in blood plasma of Control and CVD cohorts.

	Control	ACS (*p-value*)	SAP (*p-value*)	IS (*p-value*)
*n*	52	74	78	22
CE (µM)	4805 ± 973	3822 ± 1015 (<*0.0001*)	3996 ± 1040 (<*0.0001*)	4503 ± 1004 (*0.23*)
Chol (µM)	1280 ± 258	1237 ± 377 (*0.45*)	1262 ± 415 (*0.76*)	1614 ± 347 (<*0.0001*)
LPC (µM)	169 ± 53	102 ± 34 (<*0.0001*)	111 ± 29 (<*0.0001*)	127 ± 36 (<*0.0001*)
PC (µM)	2042 ± 568	1533 ± 381 (<*0.0001*)	1691 ± 396 (*0.0001*)	1840 ± 416 (*0.09*)
CE/Chol ratio	3.77 ± 0.29	3.15 ± 0.47 (<*0.0001*)	3.26 ± 0.54 (<*0.0001*)	2.83 ± 0.46 (<*0.0001*)
PC/LPC ratio	12.87 ± 4.14	16.70 ± 7.46 (*0.0002*)	15.92 ± 4.15 (<*0.0001*)	15.39 ± 4.59 (*0.03*)

Mean values (±sample standard deviations) are given for the concentrations (in µmoles/L) of a select group of blood plasma lipids (cholesteryl esters (CE), free cholesterol (Chol), lysophosphatidylcholines (LPC), and phosphatidylcholines (PC)), as well as for the concentration ratios of CE/Chol and PC/LPC. The *p*-value relative to the control is given for each case.

Compared to the Control cohort, all CVD cohorts show a very significant decrease in the concentrations of LPC. The ACS and SAP cohorts show significantly lower CE and PC concentrations which the IS cohort does not, while the IS cohort shows significantly lower Chol concentrations which the ACS and SAP cohorts do not. A more interesting aspect of the data in Table 1 is the value of the CE/Chol concentration ratio which is very significantly lower in all three CVD cohorts than it is for the Control cohort. It is also noteworthy that the sample standard deviation for the Control group is just below 8% of the average value while it is above 16% for SAP, ACS and IS groups. Within the Control cohort, the low value of the sample standard deviation for the CE/Chol concentration ratio must be compared to the typical sample standard deviations of between 20 and 33% of the average values for the concentrations for all the lipids shown (and for all other lipids measured, data not shown). This suggests that the CE/Chol ratio is very tightly controlled in the Control cohort irrespective of the large differences in the individual total cholesterol concentrations. The esterification of Chol to CE in pre-β HDL particles, catalyzed by Lecithin-Cholesterol-Acyl-Transferase (LCAT), is the only interconversion that occurs between these two lipids in the blood and any perturbation of the activity of this enzyme could be manifested in the values as well as in the sample standard deviations of the ratio. These observations led us to examine the lipidomic results for evidence of a change in LCAT activity in the CVD-cohorts. Earlier work^17^ had shown that reduced concentrations of LPC in the blood plasma were correlated with the risk for cardiovascular disease but no specific conclusions regarding possible physiological causes for the observation were drawn.

### CE concentrations are strongly correlated with the corresponding PC subspecies concentrations in plasma

**Figure 1:**
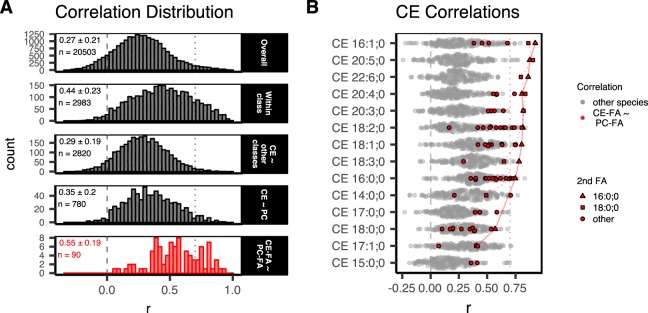
Lipid concentration correlations within the plasma lipidome. Pearson correlation coefficients (r) were calculated from the molar amounts of all lipid species (*n* = 203) across all subjects (*n* = 227). (**A**) Distribution of correlation coefficients: *Overall*: for all lipid species; *Within class*: for all species within the same class; *CE ~ other classes*: between all CE subspecies and non-CE species; *CE ~ PC*: between CE and PC lipid subspecies; and *CE-FA ~ PC-FA*: between CE and PC lipid subspecies which share a common fatty acid. Distribution’s mean, standard deviation, and *n* are indicated in the upper left corner of each panel. (**B**) Correlation coefficient (*r*) between individual CE subspecies and non-CE species (“other species” in grey). PC lipid subspecies which contain the same fatty acid (1^st^ FA) as the indicated CE subspecies are shown in red (*CE-FA ~ PC-FA*, see Table S2). CE subspecies are sorted from top to bottom according to the highest of these *CE-FA ~ PC-FA correlations*. CE subspecies, which do not match a corresponding PC subspecies have been omitted (n = 1). The second fatty acid (2^nd^ FA) of PCs, not shared with CE, is indicated by triangles in the case of 16:0;0 (connected for different CE by a solid line) and by squares in the case of 18:0;0 (connected for different CE by a dotted line). Grey dashed line indicates *r* = 0 and the grey dotted line indicates the cut-off for the correlation network shown in Fig. 2.

Pearson’s correlation coefficients (*r*) express the linear relation between variables (Fig. S2). As biosynthetic enzymes usually act upon a group of lipid species sharing similar properties (e.g. headgroup, fatty acids) rather than on a single molecular lipid species, positive correlations coefficients between the concentrations of lipid species within such groups are expected. We calculated Pearson’s *r* between all lipid subspecies over all cohorts (Fig. 1A, top panel, ${\bar{r}}_{Overall}=0.27$) and found that most lipid subspecies have positive correlations between each other, while negative correlations are a minor fraction. Notably, within-class correlations (about 15% of all correlations between subspecies belonging to the same lipid class) are enriched in positive *r* values – higher positive correlations (Fig. 1A, 2nd panel from the top, ${\bar{r}}_{{\rm{within}}-{\rm{class}}}=0.44$). This reflects that many lipid metabolism enzymes show lipid headgroup specificity^18^. The remaining 85% of cross-class correlations (a group of correlations between subspecies from different classes) expectedly result in a distribution of *r* values (${\bar{r}}_{{\rm{cross}}-{\rm{class}}}=0.25$) very similar to the original overall distribution. A fraction of cross-correlations between CE and all non-CE lipid species, as well as a group of correlations between CE and PC subspecies give both similar *r* value distributions (${\bar{r}}_{{\rm{CE}}\sim {\rm{other}}\mathrm{classes}}=0.29$ and ${\bar{r}}_{{\rm{CE}}\sim \mathrm{PC}}=0.35$ respectively, Fig. 1A, 3^rd^ and 4^th^ panels from the top), comparable to the above-mentioned cross-class correlations. However, within the set of CE ~ PC subspecies correlations, we noted a group of very positive correlations between CEs and PCs sharing a common fatty acid (CE-FA ~ PC-FA; e.g. CE 16:1;0 and PC 16:0;0_16:1;0) (Fig. 1A, bottom panel, with ${\bar{r}}_{{\rm{CE}}-{\rm{FA}}\sim {\rm{PC}}-\mathrm{FA}}=0.55$). Some of these correlation coefficients are among the highest cross-class correlations, only second to correlation coefficients between TAG and DAG species (not shown).

CEs with at least one double bond and 16, 18, 20 and 22-carbon atom-long fatty acids (this group includes the most abundant CE subspecies, except CE 16:0;0, see Fig. S1C), show the highest correlations within the CE-FA ~ PC-FA group (Fig. 1B). Each of the PC subspecies correlating to these CEs has 2 fatty acids, the *sn*-position of which cannot be discriminated in our analysis. One of the fatty acids in this group is, per definition, shared with the CE subspecies. 19 other fatty acids (occupying the other *sn*-position) were recorded (Fig. S1A, Table S2). Interestingly, PCs having 16:0;0 or 18:0;0 as a second fatty acid usually score the highest correlations within the CE-FA ~ PC-FA group (Fig. 1B).

CEs with 18:0;0 and saturated fatty acids show lower correlation coefficients and in general are not common within CE subspecies, while being much more abundant in PCs. 16:0;0 fatty acids are an exception, however, they also are much more abundant in PC than in CE (Fig. S1C).

**Figure 2:**
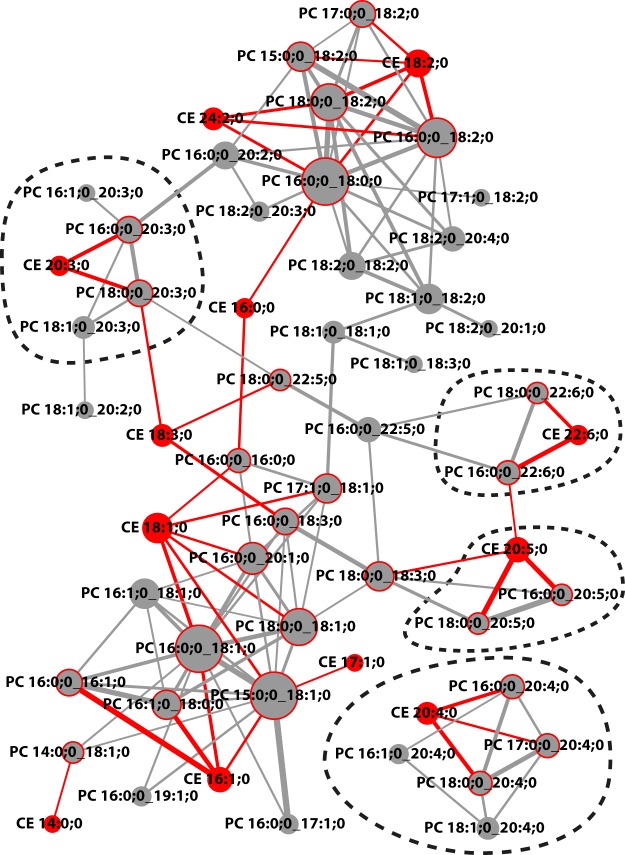
Correlation network of CE and PC lipid subspecies with cutoff *r* = 0,7 (as indicated in Fig. 1B). Node fill colors show PCs in grey and CEs in red. Node size is adjusted to the number connections. Red node border colors in PC nodes indicate a connection to a CE. Edge color is grey for PC-PC connections and red if a CE is involved. Edge stroke widths indicate the magnitudes of the correlation coefficient. Dashed lines encircle clusters sharing the same fatty acids.

We used the data to create a correlation network between PC and CE subspecies, which reveals distinct clusters, reflecting the organization of co-regulated subspecies (Fig. 2). Clusters of lipids containing 20:3;0, 20:4;0, 20:5;0 and 22:6;0 fatty acids are distinguishable, while lipids with 16 and 18 carbon atoms are more interconnected.

In summary, we find very high correlations between PC and CE subspecies sharing a common fatty acid which is in agreement with LCAT activity in blood plasma.

### Conversion of Chol to CE is less efficient in the blood plasma of CVD patients

The homeostasis of lipids is quite complex but may be simplified by considering that their concentrations in the blood plasma compartment result from the exchange between this compartment and the tissues in contact with it. The entire process may, therefore, be thought of as a classical chemical partition between two compartments so that we may write:

1 $${[{\rm{CE}}]}_{p}\rightleftharpoons {[{\rm{CE}}]}_{t},\,{\rm{with}}\,{Q}_{{\rm{CE}}}=\frac{{[{\rm{CE}}]}_{t}}{{[{\rm{CE}}]}_{p}}\,{\rm{or}}\,{[{\rm{CE}}]}_{t}={Q}_{{\rm{CE}}}{[{\rm{CE}}]}_{p};$$

2 $${[{\rm{LPC}}]}_{p}\rightleftharpoons {[{\rm{LPC}}]}_{t},\,{\rm{with}}\,{K}_{{\rm{LPC}}}=\frac{{[{\rm{LPC}}]}_{t}}{{[{\rm{LPC}}]}_{p}}\,\,{\rm{or}}\,{[{\rm{LPC}}]}_{t}={K}_{{\rm{LPC}}}{[{\rm{LPC}}]}_{p};$$

3 $${[{\rm{Chol}}]}_{p}\rightleftharpoons {[{\rm{Chol}}]}_{t},\,{\rm{with}}\,{Q}_{{\rm{Chol}}}=\frac{{[{\rm{Chol}}]}_{t}}{{[{\rm{Chol}}]}_{p}}\,{\rm{or}}\,{[{\rm{Chol}}]}_{t}={Q}_{{\rm{Chol}}}{[{\rm{Chol}}]}_{t};$$

4 $${[{\rm{PC}}]}_{p}\rightleftharpoons {[{\rm{PC}}]}_{t},\,{\rm{with}}\,{Q}_{{\rm{PC}}}=\frac{{[{\rm{PC}}]}_{t}}{{[{\rm{PC}}]}_{p}}\,\,{\rm{or}}\,{[{\rm{PC}}]}_{t}={Q}_{{\rm{PC}}}{[{\rm{PC}}]}_{p};$$

where the boxed brackets refer to concentrations, the subscripts *p* and *t* refer to the plasma and tissue compartments, respectively. *Q*_CE_, *Q*_Chol_, and *Q*_PC_ are the non-equilibrium reaction quotients for the partition processes that are not expected to attain a rapid equilibrium and include partition processes that are mediated by transport mechanisms as well as non-mediated passive processes; and *K*_LPC_ is the equilibrium partition coefficient for LPC between the plasma and tissue compartments. The rapid insertion, and desorption of LPC into/from lipid bilayers and similar amphiphilic surfaces^19^ justifies the use of an equilibrium partition coefficient in this case since it is expected to attain equilibrium in minutes through passive, non-mediated processes. The extremely rapid kinetics for passive partitioning of LPC^19^, between blood plasma components and membranes of cells in contact with the plasma, compared with PC^20,21^ and Chol^22,23^ also makes the concentration of LPC in the plasma compartment relatively low compared with that of the other lipids of interest. On the other hand, it is probably safe to assume that partitioning of CE between the plasma and the tissues is exclusively mediated by transport mechanisms due to its hydrophobicity and location in the interior of emulsion-like particles (lipoproteins in plasma and lipid droplets in cells).

We may now write the PC-dependent conversion of Chol to CE in the entire system as:

$${\rm{C}}{\rm{h}}{\rm{o}}{\rm{l}}+{\rm{P}}{\rm{C}}\rightleftharpoons {\rm{C}}{\rm{E}}+{\rm{L}}{\rm{P}}{\rm{C}},$$

for which the global non-equilibrium reaction quotient at any given point for any given individual is given by:

5 $$Q=\frac{{[{\rm{C}}{\rm{E}}]}_{T}.{[{\rm{L}}{\rm{P}}{\rm{C}}]}_{T}}{{[{\rm{C}}{\rm{h}}{\rm{o}}{\rm{l}}]}_{T}.{[{\rm{P}}{\rm{C}}]}_{T}},$$

where the subscript *T* indicates the total concentration. For any given compound under consideration,

$${[{\rm{X}}]}_{T}=\frac{{{\rm{V}}}_{p}{[{\rm{X}}]}_{p}}{{{\rm{V}}}_{T}}+\frac{{{\rm{V}}}_{t}{[{\rm{X}}]}_{t}}{{{\rm{V}}}_{T}},$$

where V are the compartment volumes, and X may be Chol, CE, PC, or LPC. Thus:

$$Q=\frac{({{\rm{V}}}_{p}{[{\rm{CE}}]}_{p}+{{\rm{V}}}_{t}{[{\rm{CE}}]}_{t}).({{\rm{V}}}_{p}{[{\rm{LPC}}]}_{p}+{{\rm{V}}}_{t}{[{\rm{LPC}}]}_{t})}{({{\rm{V}}}_{p}{[{\rm{Chol}}]}_{p}+{{\rm{V}}}_{t}{[{\rm{Chol}}]}_{t}).({{\rm{V}}}_{p}{[{\rm{PC}}]}_{p}+{{\rm{V}}}_{t}{[{\rm{PC}}]}_{t})},$$

which, substituting the values for [X]_*t*_ from Equations 1–4, gives:

$$Q=\frac{\{{[{\rm{CE}}]}_{p}.({{\rm{V}}}_{p}+{{\rm{V}}}_{t}{Q}_{{\rm{CE}}})\}.\{{[{\rm{LPC}}]}_{p}.({{\rm{V}}}_{p}+{{\rm{V}}}_{t}{K}_{{\rm{LPC}}})\}}{\{{[{\rm{Chol}}]}_{p}.({{\rm{V}}}_{p}+{{\rm{V}}}_{t}{Q}_{{\rm{Chol}}})\}.\{{[{\rm{PC}}]}_{p}.({{\rm{V}}}_{p}+{{\rm{V}}}_{p}{Q}_{{\rm{PC}}})\}},$$

which may be re-written:

6 $$Q^{\prime} =Q\frac{({{\rm{V}}}_{p}+{{\rm{V}}}_{t}{Q}_{{\rm{Chol}}}).({{\rm{V}}}_{p}+{{\rm{V}}}_{t}{Q}_{{\rm{PC}}})}{({{\rm{V}}}_{p}+{{\rm{V}}}_{t}{Q}_{{\rm{CE}}}).({{\rm{V}}}_{p}+{{\rm{V}}}_{t}{K}_{{\rm{LPC}}})}=\frac{{[{\rm{CE}}]}_{p}.{[{\rm{LPC}}]}_{p}}{{[{\rm{Chol}}]}_{p}.{[{\rm{PC}}]}_{p}}.$$

The right-hand side of Eqn. 6 is the reaction quotient for the LCAT-catalyzed conversion of Chol to CE in the plasma compartment and its exact value can be calculated from the plasma lipidomic data. *Q*′ is the reaction quotient for the global conversion of Chol to CE using PC as the fatty acid source. Its relationship to the LCAT-catalyzed reaction quotient in the plasma is conditioned by the non-equilibrium partition quotients, *Q*_CE_, *Q*_Chol_, and *Q*_PC_, as well as the equilibrium constant, *K*_LPC_ since the LCAT reaction and the partition processes are connected to each other. Given that *K*_LPC_, describes a non-mediated partition process that is in rapid equilibrium, it can be safely assumed to be a constant. The tissue and plasma volumes V_*t*_ and V_*p*_ can be also assumed to be constants for any given individual.

In principle, the value of *Q*′ provides a global index of cholesterol homeostasis as manifested in the blood plasma compartment of any given individual. It is based upon a relatively simple and straightforward analysis of lipid concentrations in the blood plasma. It includes tissue/plasma partition processes for Chol, CE, and PC, and LPC, and the conversion of Chol to CE in the blood catalyzed by LCAT. It is, strictly speaking, not an exclusive measure of LCAT activity. However, if it can be assumed that there are no significant CVD-related functional differences between the CVD and Control cohorts with regard to the mediated “partitioning” of CE, Chol and PC between the tissue and plasma compartments, *Q*′ becomes an exact measure of the LCAT-catalyzed conversion of Chol to CE, at the expense of PC and producing LPC, in the plasma compartment.

**Figure 3:**
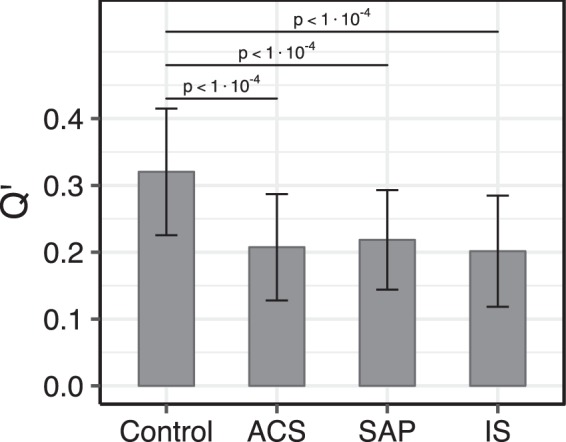
The figure shows the reaction quotient *Q*′, (with standard deviations) for the Control and CVD cohorts. Comparison of the distributions of the control and pathological states gave two-tailed *p*-values as indicated, while comparisons between pathological states gave *p*-values > 0.4.

From the lipidomic data we have calculated the value of *Q*′ for all the cohorts examined. The results are shown graphically in Fig. 3. The values of *Q*′ in the CVD cohorts (*Q*′_ACS_ = 0.217 ± 0.084; *Q*′_SAP_ = 0.220 ± 0.071; and *Q*′_IS_ = 0.202 ± 0.082) are all about 2/3 of the value of *Q*′ in the control cohort (*Q*′_Control_ = 0.320 ± 0.095). The differences in *Q*′ values between the control and CVD cohorts are highly significant, with two-tailed *p*-values < 1 × 10^−4^ (Fig. 3 and Supplementary Table S3). There are no differences in the value of *Q*′ between the acute phase (ACS and IS) and non-acute phase (SAP) cohorts, or related to prior statin use of the donor within the same cohort (Table S3).

It should be emphasized that the values of *Q*′ provide a better index of global cholesterol homeostasis as manifested in the blood plasma than simple measurements of total cholesterol (Chol + CE), whether in whole plasma or sub-fractions thereof, because they include the exchange of cholesterol and its esters between the tissue and the blood plasma compartments as well as their physiological-chemical interconversions, the LCAT-catalyzed reaction, within the plasma compartment.

We applied a series of logistic regressions classifying patients using *Q*′, its components individually, and all components, including *Q*′, combined (Table S3). *Q*′ was found to be highly significant in all cases, as were many of its components. Prediction metrics show *Q*′ to have highly predictive scores, only slightly superseded by LPC in the cases of ACS and SAP. When all component plus *Q*′ were used in a multivariate model the scores were even better than the individual components. In the latter case, only CE and Chol remain significant. However, *Q*′ as a predictor is preferable since it gives an additional mechanistic insight.

### *Q*′ is related to the conversion of pre-βHDL to HDL_3_ in the blood plasma

**Figure 4:**
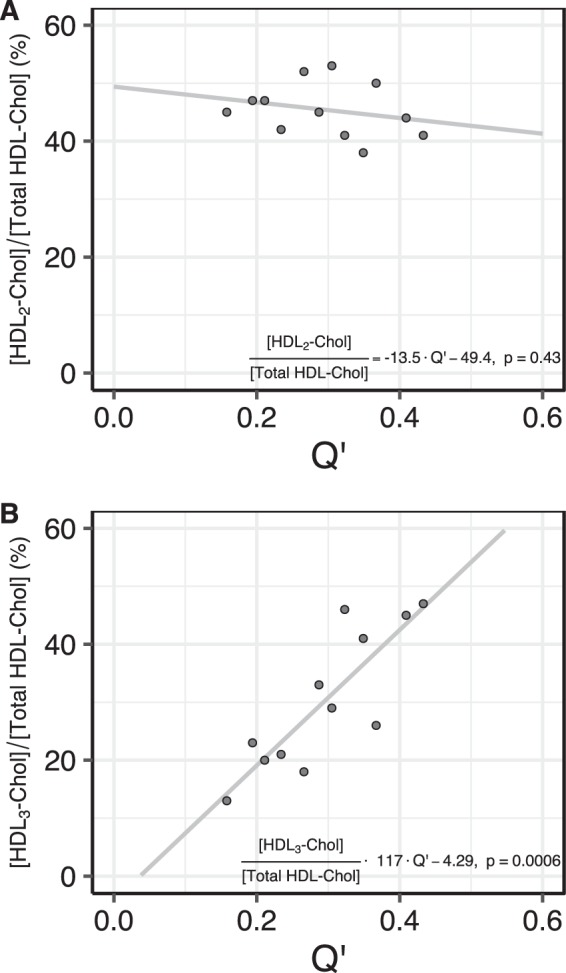
The relationship of the reaction quotient *Q*′ to the fraction of total HDL-Chol that is found in the HDL_2_ (panel A) and HDL_3_ (panel B) sub-fractions.

We also enquired how *Q*′ is related to the cholesterol content of the HDL_3_ and HDL_2_ sub-fractions of the HDL fraction. In principle, a deficiency in the LCAT activity should manifest itself as a change in the fraction of total cholesterol in the various HDL sub-fractions. Specifically, since LCAT acts preferentially upon the HDL fraction of lipoproteins in the blood and catalyzes conversion of the pre-β HDL (discoid particles) to HDL_3_ (small, dense spherical particles) it may be expected that there should be a direct relationship between the value of *Q*′ and the fraction of the total HDL-Chol that is in the HDL_3_ sub-fraction. Figure 4 shows that while HDL_2_-Chol is not significantly related to *Q*′, HDL_3_-Chol is directly proportional to the value of *Q*′. Thus, lower values of *Q*′ also imply a less efficient conversion of Chol to CE by LCAT in the HDL fraction of lipoproteins, and consequent blockage of pre-β HDL conversion to HDL_3_ in the blood plasma.

LCAT is also known to act upon lipoproteins other than HDL, notably LDL, albeit with considerably lower efficiency. The consequences of this part of the LCAT activity was not investigated in this work.

## Discussion

A general correlation analysis reveals particularly high correlations between CE and PC sharing a common fatty acid. We interpret it as a signature specific for the reaction catalyzed by the LCAT enzyme, which transfers fatty acids from PC to free cholesterol to form CE subspecies. These CE-FA ~ PC-FA correlation coefficients are among the highest cross-class correlations within the data set, only second to the correlations between the TAGs and DAGs which also can be enzymatically interconverted.

When the concentrations of the different CEs are compared with those of the other lipid species, the Pearson’s *r* values are highest for CE/PC subspecies with fatty acids of 16, 18, 20, and 22 carbon atoms having at least one double bond. These, together with the 16:0;0 fatty acid, are the acyl chains of the major CE subspecies in human plasma (ref.^24^; and Fig. S1). On the other hand, CE 16:0;0 and CE 18:0;0 score lower amongst CE-FA ~ PC-FA correlation coefficients. This result can be understood in the context of the positional specificities of the LCAT-catalyzed transfer of fatty acids from PC to Chol, as well as the positional specificities of saturated and unsaturated fatty acids in PCs. LCAT primarily transfers the fatty acid from the *sn*-2 position of PC to cholesterol but may transfer the fatty acid from the *sn*-1 position if it is poly-unsaturated^25,26^, while, as observed in human erythrocytes, the positional specificity of fatty acids in PC subspecies overwhelmingly favors a saturated acyl chain (16:0 or 18:0) in the *sn*-1 position and an unsaturated acyl chain in the *sn*-2 position^27^. Thus PCs containing 16:0;0 and 18:0;0 fatty acids, are likely to have these fatty acids in the *sn*-1 position, therefore are not transferred by LCAT and thus have lower correlation coefficients. As our lipidomic method cannot distinguish between the *sn*-1 and *sn*-2 positions, we are likely to observe lower correlation coefficients than if positional isomerism could be distinguished. Altogether, our results support the thesis that the CE-FA ~ PC-FA correlation coefficients can be explained by LCAT activity.

Epidemiological studies have clearly demonstrated a robust negative correlation between high levels of HDL-cholesterol and CVD risk but the exact reasons why this is the case are not clear. The biochemistry of HDL in the blood, unlike that of the other lipoprotein particles, is quite complex since it involves both influx and efflux of components (which is common to all lipoproteins) as well as their chemical interconversion in the plasma (which occurs mostly in the HDL). After acquiring excess cholesterol and some PC from the peripheral tissue cells, discoid pre-β HDL particles are subjected to the enzymatic activity of LCAT that converts Chol to CE at the expense of the PC component of the same particle. The highly apolar CE segregates into the bilayer midplane of the discoid HDL which, consequently, assume a progressively more spherical shape. These small, dense spherical particles (HDL_3_ sub-fraction) fuse to form larger, less dense spherical particles (HDL_2_ sub-fraction). The HDL_3_ sub-fraction exchanges its CE for triglycerides from other lipoprotein particles, a process mediated by the serum cholesteryl ester transfer protein, or releases its apolar (CE and triglyceride) content to the liver via a receptor mediated process^8,28^. This complexity has led to the suggestion that “cardiovascular risk management may be more accurate if risk assessment relies on more precise measurements of HDL metabolism than HDL-C” and “it might also be useful to develop novel biomarkers that actually provide information about HDL functionality”^5^, and that “a major clinical challenge is to link molecular mechanisms underlying HDL’s cardioprotective activities to robust, quantitative metrics that can be widely applied in translational and clinical studies”^7^. The present work is a step in that direction and shows that the value of *Q*′ is, on the average, reduced by a third in the CVD cohorts compared with the Control cohort (Fig. 3). The results suggest a strong diagnostic/predictive value of this parameter for atherosclerosis-related CVD. This will have to be confirmed by a prospective cohort study in the future.

The role of LCAT in atherosclerosis is highly controversial^29,30^. In principle, there are at least three factors that can influence the value of *Q*′ in blood plasma: (1) The concentration of LCAT in CVD cohorts could be lower than it is in the control cohort. There is, however, very good evidence that this is not the case^31^; (2) The conversion of Chol to CE (the LCAT-catalyzed reaction, without involving a decrease in LCAT concentration) could be less efficient in the plasma of patients with atherosclerosis; (3) The Chol efflux from peripheral tissues to HDL, and therefore the concentration of Chol in the plasma, could be negatively correlated with the amount of atherosclerosis^32,33^. Points (2) and (3) above are interrelated in complicated ways. On the one hand, a reduced cholesterol efflux capacity of HDL would lower its concentration for the subsequent LCAT reaction in the plasma and therefore reduce its apparent efficiency. On the other hand, a reduced capacity of LCAT to convert Chol to CE would also lead to an accumulation of Chol in the pre-β HDL sub-fraction and mass action would reduce Chol efflux from the peripheral cell compartment to the plasma (specifically, the HDL) compartment. The lipidomic analysis (Table 1) indicates that while CE concentrations are lower in the plasma of the CVD cohorts, Chol concentrations are not. It is possible that the apparent lower cholesterol efflux from macrophage cultures *in vitro* provoked by Apo-B-depleted plasma from CVD patients^32^ is a consequence of the discoid HDL in this plasma being already saturated with Chol (for the limits of solubility of Chol in phospholipid bilayers see^34^), resulting from the lack of its conversion to CE due to an inefficient LCAT-catalyzed reaction.

We might, therefore, enquire why the efficiency of the LCAT reaction is negatively correlated with CVD. One possibility is that the pre-β HDL contains some of the products resulting from oxidation of polyunsaturated fatty acids (PUFAs) that are attached to the PC^35,36^ or to cholesterol in CEs^37,38^ in LDL trapped within the arterial intima. PC-hydroperoxides have been shown to be competitive inhibitors of LCAT^35^. These compounds might be expected to be scavenged by small HDL particles in the blood plasma and, due to their structural similarities with the substrates of LCAT, act as inhibitors of LCAT. If this thesis is accepted, it would mean that pre-β HDL do not progress efficiently to the HDL_3_ spherical forms in the plasma of CVD patients, confirming earlier findings^39–41^ and this would imply an effective blocking of reverse cholesterol transport. Our results indicate that *Q*′, obtained from lipidomic analyses of blood plasma, may be a good measure of this inefficiency.

There has been much debate in the literature as to whether the clearly established inverse correlation of HDL-cholesterol levels with atherosclerosis risk is causative or consequential^21,29,42–46^. Our results indicate that there is an inhibition of the LCAT-catalyzed conversion of cholesterol to its esters and consequent blockage of pre-β HDL conversion to HDL_3_ in the blood of CVD patients (whether in the acute phase (ACS and IS) or not (SAP)). This inhibition/blockage results in a saturation of the capacity of pre-β HDL to participate in cholesterol efflux from the tissue to the blood plasma. HDL-cholesterol levels would, therefore, appear to be a consequence of atherosclerosis and not its cause.

## Materials and Methods

### Plasma samples

Blood samples were obtained from 4 groups of donors: The first group (52 cases, 34 women and 18 men, ages 36–82 years), labeled “Control”, were randomly taken from the population of the Coimbra and Lisbon, Portugal, regions who satisfied the criterion that they had never had any CVD-related health complaints. The second group (74 cases, 18 women and 56 men, ages 36–92 years), labeled “ACS”, was composed of patients admitted at the Hospital Santa Cruz, Carnaxide, Portugal, who suffered ST-segment elevation myocardial infarction (21 cases, 4 women and 17 men, ages 37–82 years), Non-ST-segment elevation myocardial infarction (38 cases, 10 women and 28 men, 36–92 years) or unstable angina (15 cases, 4 women and 11 men, 50–87 years). The third group, labeled “SAP” (78 cases, 22 women and 56 men, ages 36–89 years,) was composed of patients admitted at the Hospital Santa Cruz, Carnaxide, Portugal, suffering from stable angina pectoris. The fourth group (Ages 21 cases, 14 women and 7 men, ages 49–94 years), labeled “IS” was composed of patients admitted at the emergency room of the Centro Hospitalar de Lisboa Ocidental, Lisbon, Portugal, who suffered from acute ischemic stroke. For purposes of this report, we will consider the groups ACS, SAP and IS to be suffering from cardiovascular disease (CVD). In the case of the ACS and IS cohorts blood was drawn within the first 24 h of admission into the hospital emergency rooms. In the SAP cohort blood was drawn on the occasion of routine medical examination at the hospital.

Blood was obtained from all donors after explaining the purpose and obtaining written informed-consent from them or their legal representatives. The entire process was approved by the Ethical Review Board of the Faculty of Medicine of the New University of Lisbon and the Ethics Committee for Health of the Centro Hospitalar de Lisboa Ocidental, that includes the Hospital Santa Cruz, the Hospital Egas Moniz and Hospital São Francisco Xavier and all experiments were performed in accordance with the guidelines and regulations. Blood samples were drawn into tubes containing an anti-coagulant (heparin or EDTA) immediately after admission into the hospital and signing of the informed-consent. The samples were kept at 4 °C and processed within 24 h from collection. Plasma was obtained by centrifugation of the blood at 500 g for 10 min at 4 °C, frozen at −80 °C and stored at this temperature until they were used for the lipidomic analysis. Total Chol, HDL_2_-Chol and HDL_3_-Chol was determined by the laboratory for clinical analysis: Germano de Sousa – Centro de Medicina Laboratorial, Lisbon.

### Lipid extraction for mass spectrometry lipidomics

Mass spectrometry-based lipid analysis was performed at Lipotype GmbH (Dresden, Germany) as described (Surma *et al*.^14^). For lipid extraction an equivalent of 1 µL of undiluted plasma was used. Internal standards were pre-mixed with the organic solvents mixture and included: cholesterol D6, cholesteryl ester 20:0, ceramide 18:1;2/17:0, diacylglycerol 17:0/17:0, phosphatidylcholine 17:0/17:0, phosphatidylethanolamine 17:0/17:0, lysophosphatidylcholine 12:0, lysophosphatidylethanolamine 17:1, triacylglycerol 17:0/17:0/17:0, and sphingomyelin 18:1;2/12:0. All liquid handling steps were performed using Hamilton Robotics STARlet robotic platform with the Anti Droplet Control feature for organic solvents pipetting.

### MS data acquisition

Samples were analyzed by direct infusion in a QExactive mass spectrometer (Thermo Scientific) equipped with a TriVersa NanoMate ion source (Advion Biosciences). Samples were analyzed in both positive and negative ion modes with a resolution of R_m/z=200_ = 280000 for MS and R_m/z=200_ = 17500 for MSMS experiments, in a single acquisition. MSMS was triggered by an inclusion list encompassing corresponding MS mass ranges scanned in 1 Da increments. Both MS and MSMS data were combined to monitor CE, DAG and TAG ions as ammonium adducts; PC, PC O-, as acetate adducts; and PE, PE O- and PI as deprotonated anions. MS only was used to monitor LPE as deprotonated anion; Cer, SM and LPC as acetate adducts and cholesterol as an ammonium adduct.

### Lipid nomenclature

When discussing different lipid species the following annotations are used: Lipid class-<sum of carbon atoms>:<sum of double bonds>;<sum of hydroxyl groups>, i.e. SM-34:1;2 means an SM species with 34 carbon atoms, 1 double bond and 2 hydroxyl groups in the ceramide backbone.

Lipid molecular subspecies^47^ annotation contains additional information on the exact identity of their fatty acids. For example PC 18:1;0_16:0;0 denotes a phosphatidylcholine with a 18:1;0 (18 carbon atoms, 1 double bond, 0 hydroxylation) and a 16:0;0 fatty acid. The exact position of the fatty acids in relation to the glycerol backbone (*sn*-1 or *sn*-2) cannot be discriminated. CE 18:1;0 denotes a cholesteryl ester with a 18:1;0 fatty acid.

### Data analysis and post-processing

Data were analyzed with in-house developed lipid identification software based on LipidXplorer^48,49^. Data post-processing and normalization were performed using an in-house developed data management system. Only lipid identifications with a signal-to-noise ratio >5, and a signal intensity 5-fold higher than in corresponding blank samples were considered for further data analysis. Using 8 reference samples per 96-well plate batch, lipid amounts were corrected for batch variations. Amounts were also corrected for analytical drift, if the p-value of the slope was below 0.05 with an R^2^ greater than 0.6 and the relative drift was above 5%. Median coefficient of (sub-)species variation as accessed by reference samples was 8.7%. An occupational threshold of 80% was applied to the data, keeping lipid species, which were present in at least 80% of the subjects in at least one cohort. Data were analysed with R version 3.3.3^50^ using tidyverse packages version 1.1.1^51^ and plots were created with ggplot2 version 2.2.1^52^. Networks were produced with Cytoscape version 3.5.0^53^. Concentrations of lipid classes used to calculate *Q′* (PC, LPC, CE, Chol) are determined as sums of molar values of individual lipid subspecies, which were determined by MS lipidomic analysis. Logistic regression models were fitted using the R glm() function. 5-times repeated 10-fold cross validation was implemented with the caret package^54^.

Comparative data for PC and total cholesterol from chemical analysis vs shotgun MS show that the MS methodology gives a value that is about 15% higher than the value obtained from chemical analysis.

## Electronic supplementary material

Supplementary Material

Dataset 1

Dataset 2
