# Supplementary material for: Cholesterol is Inefficiently Converted to Cholesteryl Esters in the Blood of Cardiovascular Disease Patients
Source: Sci Rep. 2018 Oct 3;8:14764. doi: 10.1038/s41598-018-33116-4 (PMC6170447; doi:10.1038/s41598-018-33116-4)
Supplement: Supplementary file 1 — Supplementary Material [file 41598_2018_33116_MOESM1_ESM.docx]

**Supplementary Material**

**Cholesterol is Inefficiently Converted to Cholesteryl Esters in the Blood of**

**Cardiovascular Disease Patients**

**Authors:** Mathias J. Gerl ^1,†^ ([gerl@lipotype.com](mailto:gerl@lipotype.com)), Winchil L.C. Vaz ^2,†^ ([winchil.vaz@nms.unl.pt](mailto:winchil.vaz@nms.unl.pt)), Neuza Domingues ^2^ ([neuza.domingues@nms.unl.pt](mailto:neuza.domingues@nms.unl.pt)), Christian Klose ^1^ ([klose@lipotype.com](mailto:klose@lipotype.com)), Michal A. Surma ^1^ ([surma@lipotype.com](mailto:surma@lipotype.com)), Júlio L. Sampaio ^1,§^ ([julio.lopes-sampaio@curie.fr](mailto:julio.lopes-sampaio@curie.fr)), Manuel S. Almeida ^3^ ([almeidams@sapo.pt](mailto:almeidams@sapo.pt)), Gustavo Rodrigues ^3^ ([Gustavo_rodrigues17@hotmail.com](mailto:Gustavo_rodrigues17@hotmail.com)), Pedro Araújo-Gonçalves ^3^ ([paraujogoncalves@yahoo.co.uk](mailto:paraujogoncalves@yahoo.co.uk)), Jorge Ferreira ^3^ ([jorge_ferreira@netcabo.pt](mailto:jorge_ferreira@netcabo.pt)), Claudia Borbinha ^4^ ([Claudiaborbinha3@gmail.com](mailto:Claudiaborbinha3@gmail.com)), João Pedro Marto ^4^ ([Joao.pedro.seabra.marto@gmail.com](mailto:Joao.pedro.seabra.marto@gmail.com)), Miguel Viana-Baptista ^4^ ([mvianabaptista@nms.unl.pt](mailto:mvianabaptista@nms.unl.pt)), Kai Simons^1^ ([simons@lipotype.com](mailto:simons@lipotype.com)), and Otilia V. Vieira ^2,^* ([otilia.vieira@nms.unl.pt](mailto:otilia.vieira@nms.unl.pt))

**Table S1**: Original data of lipid amounts in pmol. From this Pearson’s correlation coefficients were calculated.

**Table S2**: Correlations of CE subspecies with PC subspecies containing the same fatty acid (CE-FA~PC-FA, n=90)

**Table S3: Effect of prior Statin usage on Q′.**

Linear models were used to compare the values of *Q′* for the different cohorts. Only samples for which statin information was available were used. *p*-values are given for the direct comparison, *p*, or adjusted for statin use, *p* (Statin adjusted). The number of cases in each cohort, with and without statin use prior to blood sample collection were: Control (prior statin use, *n* = 6; without prior statin use, *n* = 46); SAP (prior statin use, *n* = 52; without prior statin use, *n* = 18); ACS (prior statin use, *n* = 30; without prior statin use, *n* = 34); IS (prior statin use, *n* = 6; without prior statin use, *n* = 15).

| **Cohort 1** | **Cohort 2** | ***p*** | ***p* (Statin adjusted)** |
| --- | --- | --- | --- |
| Control | SAP | 2.30E-09 | 7.10E-08 |
| Control | ACS | 2.00E-08 | 8.00E-08 |
| Control | IS | 3.80E-06 | 2.20E-06 |
| SAP | IS | 0.40 | 0.91 |
| ACS | IS | 0.45 | 0.61 |
| ACS | SAP | 0.99 | 0.80 |

**Table S4: Patient classification using logistic regression.**

Binary logistic regression models were used to estimate the significance of the slopes of *Q′* and its components and used to predict cohorts. Mean values (±sample standard deviations) of area under the receiver operating characteristic curve (AUC) and area under the precision/recall curve (AUPR) were determined using 5-times repeated 10-fold cross validation. Note that the baseline for AUC is 0.5, while baselines for the smaller cohorts are as follows: Control vs ACS: 0.42, IS vs Control: 0.29, and Control vs. SAP: 0.39. Significance codes: < 0.001: “a”, < 0.01: “b”, < 0.05: “c”, < 0.1: “d”, and < 0.1 are left blank.

|  |  | *p*-values | | | | | Metrics | |
| --- | --- | --- | --- | --- | --- | --- | --- | --- |
| Cohorts | Variables | CE | LPC | PC | Chol | *Q′* | AUC | AUPR |
| Control, ACS | CE | a |  |  |  |  | 0.74 ± 0.14 | 0.69 ± 0.08 |
|  | LPC |  | a |  |  |  | 0.85 ± 0.14 | 0.75 ±0.11 |
|  | PC |  |  | a |  |  | 0.72 ± 0.14 | 0.68 ± 0.10 |
|  | Chol |  |  |  |  |  | 0.40 ± 0.13 | 0.48 ± 0.06 |
|  | *Q′* |  |  |  |  | a | 0.82 ± 0.11 | 0.74 ± 0.08 |
|  | CE, LPC, PC, Chol, *Q′* | b | d |  | b |  | 0.94 ± 0.06 | 0.82 ± 0.05 |
| Control, IS | CE |  |  |  |  |  | 0.60 ± 0.21 | 0.63 ± 0.09 |
|  | LPC |  | b |  |  |  | 0.74 ± 0.20 | 0.70 ± 0.09 |
|  | PC |  |  |  |  |  | 0.57 ± 0.22 | 0.62 ± 0.11 |
|  | Chol |  |  |  | a |  | 0.78 ± 0.22 | 0.73 ± 0.08 |
|  | *Q′* |  |  |  |  | a | 0.84 ± 0.20 | 0.75 ± 0.08 |
|  | CE, LPC, PC, Chol, *Q′* | b |  |  | a |  | 0.93 ± 0.12 | 0.77 ± 0.07 |
| Control, SAP | CE | a |  |  |  |  | 0.73 ± 0.13 | 0.51 ± 0.14 |
|  | LPC |  | a |  |  |  | 0.83 ± 0.13 | 0.64 ± 0.11 |
|  | PC |  |  | a |  |  | 0.67 ± 0.16 | 0.46 ± 0.13 |
|  | Chol |  |  |  |  |  | 0.49 ± 0.15 | 0.38 ± 0.07 |
|  | *Q′* |  |  |  |  | a | 0.80 ± 0.11 | 0.58 ± 0.12 |
|  | CE, LPC, PC, Chol, *Q*′ | c |  |  | c |  | 0.89 ± 0.10 | 0.68 ± 0.09 |

| 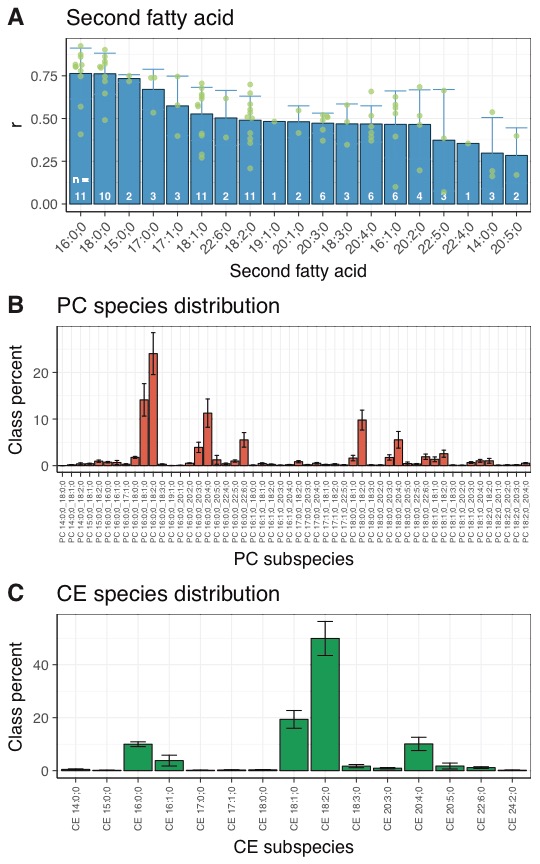 |
| --- |

**Figure S1: (A)** Mean Pearson correlation coefficients for CE-FA ~ PC-FA by second fatty acid. E.g.: For CE 18:1;0 and PC 16:0;0_18:1;0. the shared (or first) fatty acid is 18:1;0 while the non-shared (or second) fatty acid is 16:0;0. Bars show mean *r*, while points denote individual correlations. Error bars indicate standard deviations. The number of correlations (*n*) is shown at the bottom of the bars in white. **(B)** PC, and **(C)** CE subspecies distribution standardized to the lipid class. Bars indicate means over all subjects and error bars error bars indicate standard deviations.

| 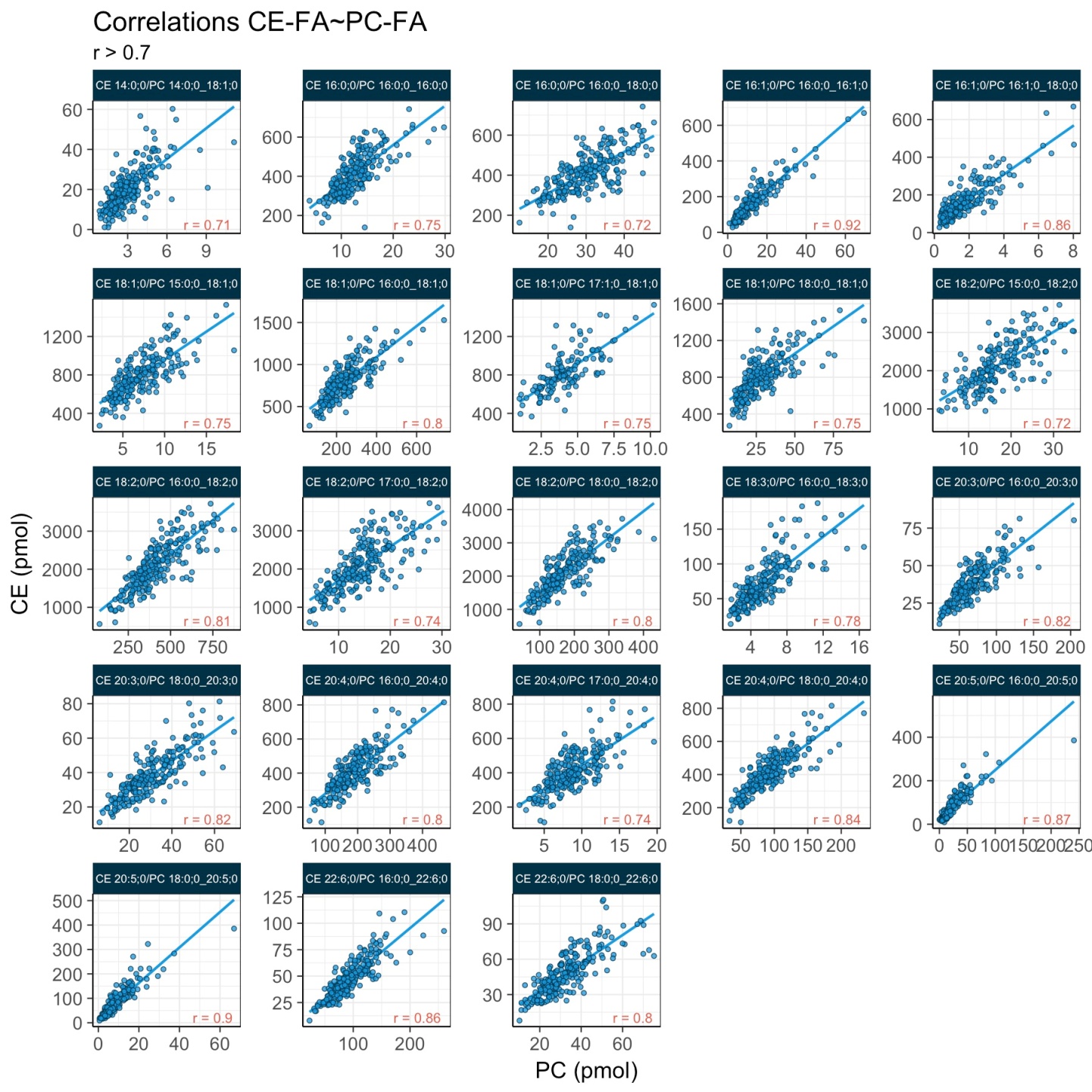 |
| --- |
| **Figure S2**: Scatter plot of subject plasma concentrations, of which the correlations of CE-FA~PC-FA are derived. Correlations with a Pearson’s coefficient greater 0.7 are shown. The correlating lipids are indicated in the strip of each panel. A linear model based on the individual points is shown as a line. The Pearson correlation coefficient (r) is indicated in each panel at the bottom right. |

**Figure S3.** Age dependence of the values of *Q’* for the “Control” cohort. Data are shown for the age group 36-82 years. A linear regression model was fitted to the data and shown as a grey line. The regression equation and p-value of the slope are shown.
